# **Recurrent Dermatofibrosarcoma Protuberance and its Management with Radical Excision and Interval Skin Grafting: A Case Report**

**Published:** 2016-01

**Authors:** Imran Ahmad, Mohd Altaf Mir, Lalit Mohan Bariar, Nishat Afroz

**Affiliations:** Department of Plastic Surgery, Aligarh Muslim University, JNMCH, AMU, Aligarh, India

**Keywords:** Dermatofibrosarcoma protuberans, Radical excision, Interval skin graft

## Abstract

Dermatofibrosarcoma protuberans (DFSP) is very rare tumor of dermis layer of skin with the incidence of only 1 case per million per year. DFSP rarely leads to a metastasis (Less than 5% have metastasis), but DFSP can recur locally. We publish a rare case of a recurrent dermatofibrosarcoma protuberans and its management with radical excision and interval skin grafting*.*

## Introduction

Dermatofibrosarcoma protuberans (DFSP)is a very rare tumor.1 It is a rare neoplasm of the dermis layer of the skin, and is classified as a sarcoma. There is only about one case per million per year. DFSP is a fibrosarcoma, more precisely a cutaneous soft tissue sarcoma. In many respects, the disease behaves as a benign tumor, but in 2–5% of cases it can metastasize, so it should be considered to have malignant potential. It occurs most often in adults in their thirties; it has been described congenitally, in children, and the elderly. It accounts for approximately 2–6% of soft tissue sarcoma cancers.^1^

Dermatofibrosarcoma protuberans is diagnosed with a biopsy, when a portion of the tumor is removed for examination. In order to ensure that enough tissue is removed to make an accurate diagnosis, the initial biopsy of a suspected DFSP is usually done with a core needle or a surgical incision. Dermatofibrosarcoma protuberans begins as a minor firm area of skin most commonly about to 1 to 5 cm in diameter. It is a slow growing tumor and is usually found on the torso but can also be found on the arms, legs, head and neck. About 90% of DFSPs are low grade sarcomas.^2^^,^^3^

About 10% are mixed; they contain a high-grade sarcomatous component; therefore, they are considered to be intermediate-grade sarcomas. DFSP rarely leads to a metastasis (fewer than 5% do metastasis), but DFSP can recur locally. DFSPs most often arise in patients who are in their thirties, but sometimes have been described in children or the elderly. More than 90% of DFSP tumors have the chromosomal translocation t(17;22). The translocation fuses the collagen gene (COL1A1) with the platelet-derived growth factor (PDGF) gene. The fibroblast, the cell of origin of this tumor, expresses the fusion gene in the belief that it codes for collagen.

However, the resulting fusion protein is processed into mature platelet-derived growth factor which is a potent growth factor. Fibroblasts contain the receptor for this growth factor. Thus the cell “thinks” it is producing a structural protein, but it actually produces a self-stimulatory growth signal. The cell divides rapidly and a tumor forms. The tissue is often positive for CD34.^2^^,^^3^

The standard of care for patients with DFSP is surgery. Usually, complete surgical resection with margins of 2 to 4 cm is recommended. The addition of adjuvant radiotherapy improves local control in patients with close or positive margins during the surgery. Patients who have a recurrent DFSP can have further surgery, but the probability of adverse effects of surgery and/or metastasis is increased in these patients. Imatinib is approved for treatment. Imatinib is a small molecular pathway inhibitor and inhibits tyrosine kinase. It may be able to induce tumor regression in patients with recurrent DFSP, unresectable DFSP or metastatic DFSP. There is clinical evidence that imatinib, which inhibits PDGF-receptors, may be effective for tumors positive for the t(17;22) translocation.^2^^,^^3^

## CASE REPORT

**Fig. 1:**
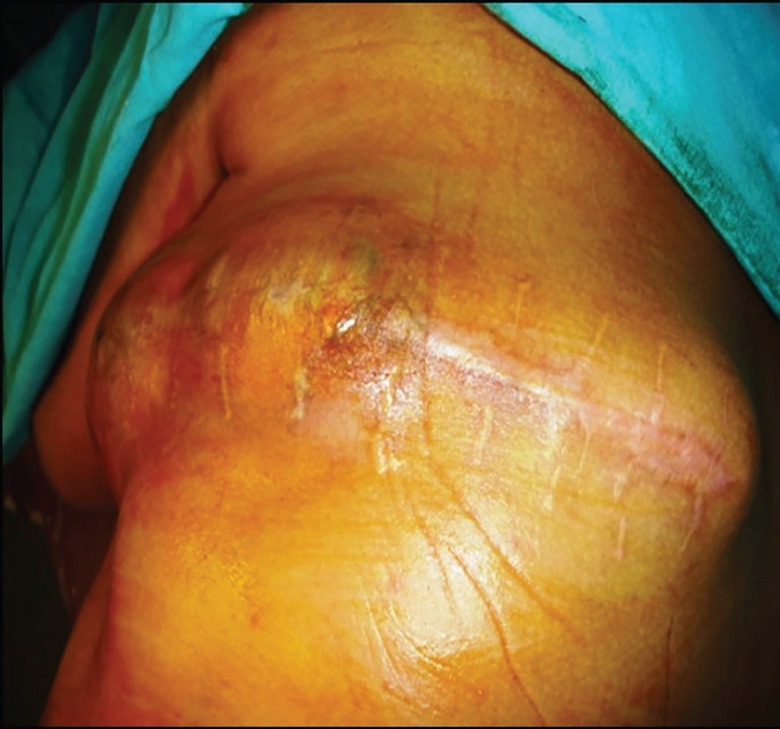
Pre-operation image of lesion

In the year 2014, a 60 year old male from a Sikh community presented to Plastic Surgery OPD of our institution with a huge recurrent swelling in left upper back since last 4 years. He had first noticed a 1-cm nodule on his left upper back which had grown gradually since then to attain its present size of 10 cm x 25 cm. The swelling was oval in shape with surgical scar and bossellated surface (Figure 1). He was operated twice previously. First surgery in 2012, second in 2013; but both surgeries were performed by a general surgeon.

Margin clearance had not been achieved and wound was closed primarily during all previous surgeries. Previous histopathology report was suggestive of DFSP with margin involvement. Metastatic work up was normaland there was no evidence of metastasis. Patient was operated under general anaesthesia, Radical (wide) local excision of tumour was donetaking a margin of 4cm from indurated margins. Resection included subcutaneous tissue, Latissimus dorsi (LD) muscle and part of serratus posterior muscle. The medial border of scapula was stabilised with sutures.

**Fig. 2:**
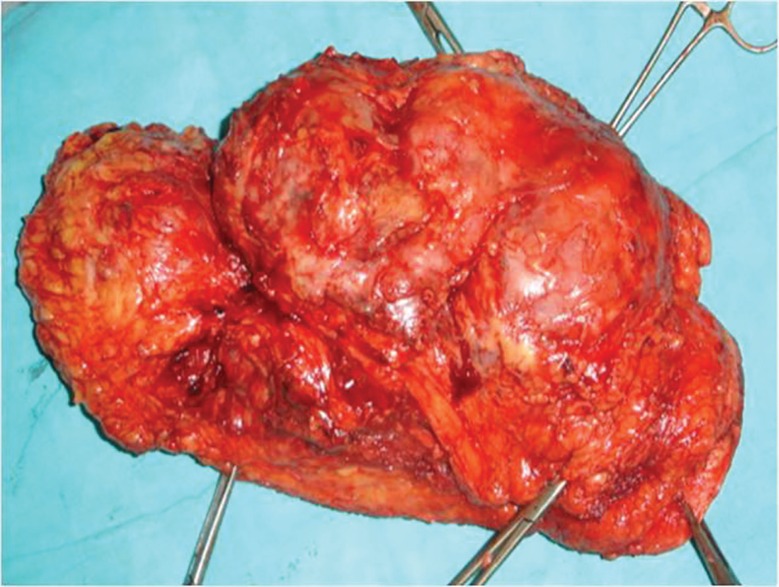
Macroscopic features of excised specimen

**Fig. 3:**
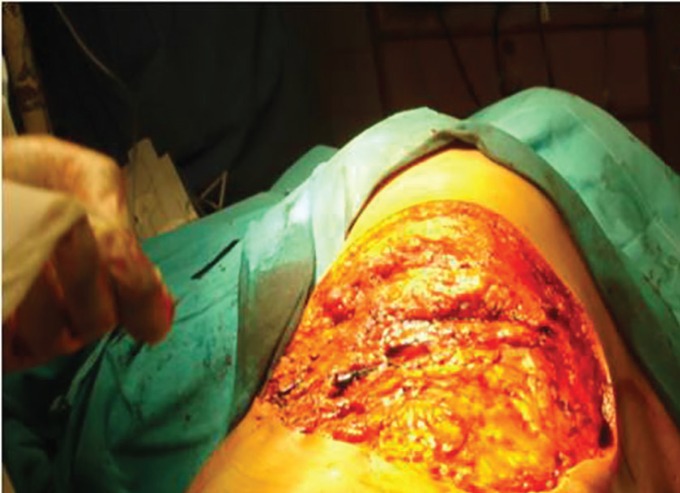
Postsurgical defect

**Fig. 4:**
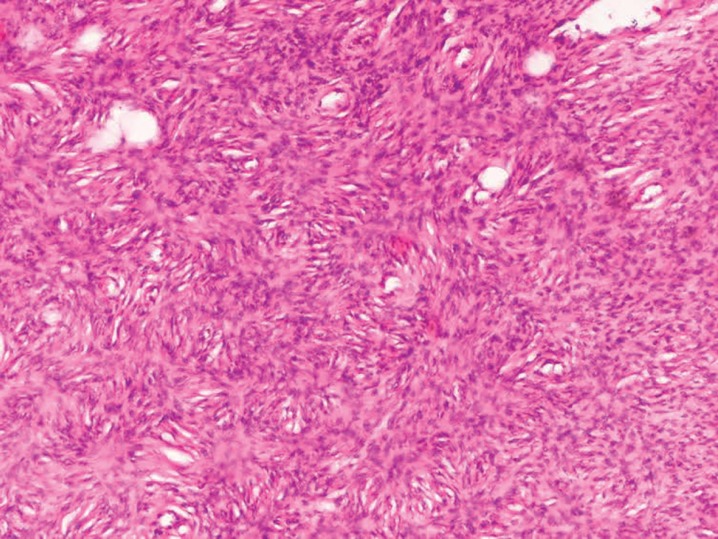
Typical cartwheel appearance of DFSP

Macroscopic examination of the excised tumor revealed potato like bulbous extensions on its surface and cut surfaces were pink-white in colour (Figure 2). The post surgical defect (Figure 3) was not covered. Haemostasis was achieved and controlled pressure dressing was done. Post op period was uneventful. On histopathological examination, surgical resection margins were found to be negative after operation. Histopathological examination confirmed fibrosarcoma (Figure 4 classical cartwheel pattern) and immunohistochemistry confirmed the CD34 positivity. 

**Fig. 5:**
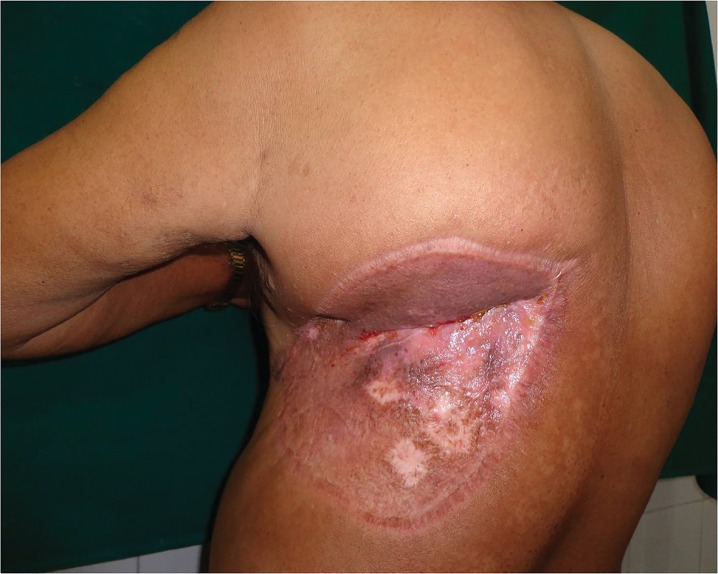
Defect after skin grafting

Skin grafting was done after 3 weeks (Figure 5). Patient was discharged two weeks after skin grafting and was referred for radiotherapy to the Department of Radiation Oncology. We followed up the patient every 2 months. The patient’s general condition was good at the last follow-up, no evidence of local relapse was seen and no enlargement of cervical and axillary lymph nodes were noted clinically. The patient was actively using his arm and was carrying out his routine daily activities without any deficit.

## DISCUSSION

Dermatofibrosarcoma protuberans was first named byHoffmann^4^ in 1925 when he reported three cases, however; Darrier and Ferrand established these interesting lesions as a distinct clinical entity in 1924.^5^ It is described by other terms like, progressive and recurrent dermatofibroma by Darrierand Ferrand,^5^ hypertrophic morpheaby Sherwell,^6^^,^^7^ sarcomatoustumor resembling keloid by Taylor,^8^ fibrosarcoma of the skin(Stout).^9^ Malignantfibrous xanthoma, was included by O’Brien and Stout^10^ in dermatofibrosarcoma protuberans. 

Dermatofibrosarcoma protuberans (DFSP)^1^ is a rare neoplasm of the dermis layer of the skin, and is classified as a sarcoma. There is only about one case per million per year. In many respects, the disease behaves as a benign tumor, but in 2–5% of cases it can metastasize. The treatment of choice is complete resection with a wide margin of surrounding tissue, including the underlying fascia. The safety margin should reach 3 to 4 cm beyond the macroscopic lesion and the margins must be histologically negative, otherwise the recurrences occur in approximately 70% of patients.

Therefore, in management of locally malignant tumours one should not be lenient in the fair of development of large post surgical defect. Thus in the management of postsurgical defects, planning in reverse is very important. Another alternative treatment is Mohs micrographic surgery, but this is usually left for specific locations such as the face or eyelids, where large resections may lead to functional problems.^11^ Mohs micrographic surgery has the advantage of high oncologic effectiveness and maximal tissue savingand is increasingly accepted as the treatment of choice.^11^

Further outpatient care is important as the tumor has a proclivity for recurrence. Poor prognosis is characterized by its late presentation, aggressive local invasion, regional nodal involvement and distant metastasis. Some histologic features serve as poor prognostic indicators; high number of mitotic figures, increased cellularity, DNA aneuploidy, TP53 gene over expression and fibrosarcomatous change.^12^

We conclude that in management of locally malignant tumorsone should not be lenient and planning in reverse is very importantand we shouldnever compromise to excise in fear of residual defect size and always consult your colleague for any help**.**

## References

[1] Mendenhall WM, Zlotecki RA, Scarborough MT Dermatofibrosarcoma protuberans. Cancer.

[2] Sirvent N, Maire G, Pedeutour F (2003). Genetics of dermatofibrosarcoma protuberans family of tumors: from ring chromosomes to tyrosine kinase inhibitor treatment. Genes Chromosomes Cancer.

[3] Patel KU, Szabo SS, Hernandez VS, Prieto VG, Abruzzo LV, Lazar AJ, López-Terrada D (2008). Dermatofibrosarcoma protuberans COL1A1-PDGFB fusion is identified in virtually all dermatofibrosarcoma protuberans cases when investigated by newly developed multiplex reverse transcription polymerase chain reaction and fluorescence in situ hybridization assays. Hum Pathol.

[4] Hoffmann E (1924). Uber das Knollentreibende Fibrosarkom der Haut (Dermatofibrosarcoma Protuberans). Dermatology.

[5] Darrier J, Ferrand M (2009). Dermatofibromes Progressifset Recidivantsou Fibrosarcomesde la Peau. Rev Med Suisse.

[6] Phelan JT, Juardo J (1963). Dermatofibrosarcoma protuberans. Am J Surg.

[7] Fisher ER, Hellstrom HR (1966). Dermatofibrosarcoma with metastases simulating hodgkin’s disease and reticulum cell sarcoma. Cancer.

[8] Taylor RW (1890). Sarcomatous tumors resembling in some respects keloids. Arch Dermatol.

[9] Stout AP (1948). Fibrosarcoma; the malignant tumor of fibroblasts. Cancer.

[10] O’Brien JE, Stout AP (1964). Malignantfibrous xanthomas. Cancer.

[11] Heuvel ST, Suurmeijer A, Pras E, Van Ginkel RJ, Hoekstra HJ (2010). Dermatofibrosarcoma protuberans: recurrence is related to the adequacy of surgical margins. Eur J Surg Oncol.

[12] Najarian DJ, Morrison C, Sait SN, Meguerditchian AN, Kane J 3rd, Cheney R, Zeitouni NC (2010). Recurrent giant cell fibroblastoma treated with Mohs micrographic surgery. DermatolSurg.

